# Three-Dimensional Segmentation of Equine Paranasal Sinuses in Multidetector Computed Tomography Datasets: Preliminary Morphometric Assessment Assisted with Clustering Analysis

**DOI:** 10.3390/s24113538

**Published:** 2024-05-30

**Authors:** Marta Borowska, Paweł Lipowicz, Kristina Daunoravičienė, Bernard Turek, Tomasz Jasiński, Jolanta Pauk, Małgorzata Domino

**Affiliations:** 1 Institute of Biomedical Engineering, Faculty of Mechanical Engineering, Białystok University of Technology, 15–351 Bialystok, Poland; p.lipowicz@pb.edu.pl (P.L.); j.pauk@pb.edu.pl (J.P.); 2 Department of Biomechanical Engineering, Vilnius Gediminas Technical University, 03224 Vilnius, Lithuania; kristina.daunoraviciene@vilniustech.lt; 3 Department of Large Animal Diseases and Clinic, Institute of Veterinary Medicine, Warsaw University of Life Sciences, 02–787 Warsaw, Poland; bernard_turek@sggw.edu.pl (B.T.); tomasz_jasinski@sggw.edu.pl (T.J.)

**Keywords:** horse, 3D segmentation, volume, surface area, K-means clustering algorithm

## Abstract

The paranasal sinuses, a bilaterally symmetrical system of eight air-filled cavities, represent one of the most complex parts of the equine body. This study aimed to extract morphometric measures from computed tomography (CT) images of the equine head and to implement a clustering analysis for the computer-aided identification of age-related variations. Heads of 18 cadaver horses, aged 2–25 years, were CT-imaged and segmented to extract their volume, surface area, and relative density from the frontal sinus (FS), dorsal conchal sinus (DCS), ventral conchal sinus (VCS), rostral maxillary sinus (RMS), caudal maxillary sinus (CMS), sphenoid sinus (SS), palatine sinus (PS), and middle conchal sinus (MCS). Data were grouped into young, middle-aged, and old horse groups and clustered using the K-means clustering algorithm. Morphometric measurements varied according to the sinus position and age of the horses but not the body side. The volume and surface area of the VCS, RMS, and CMS increased with the age of the horses. With accuracy values of 0.72 for RMS, 0.67 for CMS, and 0.31 for VCS, the possibility of the age-related clustering of CT-based 3D images of equine paranasal sinuses was confirmed for RMS and CMS but disproved for VCS.

## 1. Introduction

The paranasal sinuses comprise a bilaterally complex system of eight air-filled cavities located within the skull bones of horses [1,2,3]. This system consists of the following compartments: the frontal sinus (FS), dorsal conchal sinus (DCS), ventral conchal sinus (VCS), rostral maxillary sinus (RMS), caudal maxillary sinus (CMS), sphenoid sinus (SS), palatine sinus (PS), and middle conchal sinus (MCS), also known as the ethmoidal sinus (ES) [4,5,6]. The FS and DCS are contiguous in the horse and are sometimes collectively referred to as the conchofrontal sinus (CFS) in publications [4,5]. Similarly, the SS and PS are contiguous and are often referred to as the sphenopalatine sinus (SPS) [2,6]. Consequently, many publications describe seven compartments [6,7,8,9,10,11,12,13], while some mention six compartments [5], and others even fewer [4,14].

Some of the paranasal sinuses communicate with each other through specific apertures [4,5], while all of them connect directly or indirectly to the nasal cavity through the maxillary sinuses [6]. Consequently, the entire system is clinically significant due to its susceptibility to infections, which can spread from the nasal cavity or the alveoli of the maxillary cheek teeth [6]. As a result, diseases of the paranasal sinuses are the most common cause of unilateral nasal discharge in horses [15]. These sinus diseases can stem from multiple causes, including apical infections of the cheek teeth (dental sinusitis), benign and malignant growths within the sinuses (sinus cysts, progressive ethmoid hematomas, or sinus neoplasia), trauma (traumatic sinusitis), oro-maxillary fistulae leading to sinus feed impaction, and microbial infections (infectious sinusitis: viral, bacterial, or fungal) [12,16,17,18].

The diagnosis of paranasal sinus diseases relies on clinical examination supported by diagnostic imaging modalities, among which computed tomography (CT) allows for detailed imaging of this complex system in multiple planes without the superimposition of adjacent structures [12,19,20]. Given the anatomical complexity of the equine head, radiographic superimposition is common [8,21,22]. Therefore, CT has been employed for three-dimensional (3D) anatomical studies of the paranasal sinuses [3,4,9,10,11,13,14,23,24]. Among these studies, 3D segmentation and volumetric measurements of the paranasal sinuses have been conducted in miniature horses [13], Arabian foals [3], adult horses [14], Thoroughbred horses [9,10], and other Warmblood horses [9].

Advancements in understanding the anatomy of the equine paranasal sinus system have two practical implications. First, CT-based 3D volumetric measurements [3,9,10,13,14] can assist in developing more accurate diagnostic protocols and innovative treatment strategies, such as balloon sinuplasty [25] and CT-based presurgical planning [20,26], for horses with paranasal sinus diseases. Second, CT-generated 3D computer models can serve as effective educational tools, enhancing confidence, enjoyment, and knowledge acquisition. These models are a valuable addition to the veterinary anatomy curriculum [11]. Studies have shown that 3D models are more helpful than traditional 2D lectures for understanding the complex anatomy of the equine paranasal sinuses [11].

Thus, this study aimed to enhance the current protocols for CT-based segmentation and volumetric measurements of the equine paranasal sinuses by incorporating measurements of their surface area and relative density. Subsequently, a clustering analysis was implemented for the computer-aided identification of age-related variations in the extracted morphometric measures.

## 2. Materials and Methods

### 2.1. Study Design

This observational prospective cohort study was conducted on the heads of 18 Warmblood horses (mean body weight of horses ± SD: 590 ± 35 kg; mean height of horses at the withers ± SD: 162 ± 4 cm), comprising 10 mares and 8 geldings, of ages ranging from 2 to 25 years (mean age ± SD: 11.83 ± 7.80 years). The collected heads represented three age-related groups: young horses (2 mares and 4 geldings; ages 2 to 4 years; mean age ± SD: 2.83 ± 0.98 years), middle-aged horses (4 mares and 2 geldings; ages 11 to 12 years; mean age ± SD: 11.50 ± 0.55 years), and old horses (4 mares and 2 geldings; ages 20 to 25 years; mean age ± SD: 21.17 ± 1.94 years). Six heads were enrolled in each group. All horses were slaughtered in a commercial slaughterhouse for breeding purposes, to obtain meat. None of the horses had a known history or clinical signs of paranasal sinus disease at the time of slaughter. Immediately after slaughter, the heads were isolated and transported to the Equine Clinic at the Warsaw University of Life Sciences.

### 2.2. CT Image Acquisition

In the Equine Clinic, the isolated heads were imaged using a 64-slice CT scanner (Revolution CT, GE Healthcare, Chicago, IL, USA). The scan length was adjusted between the rostral aspect of the lips and the caudal aspect of the occipital bone, with the number of slices tailored to each head’s size. The following scanning parameters were applied: helical scan type GSI 20 mm; current 275 mA; voltage GSI–QC (Dual Energy, variable in the range of 70–140 kV) X-ray tube current; gantry rotation 0.08/s/HE+; table travel 39.4 mm/rotation; pitch 0.984:1; slice thickness 2.5 mm; and spatial resolution 0.23 mm/50 cm SFOV.

The CT scans were processed using the AW workstation (GE Healthcare, Chicago, IL, USA) with specialized software (VolumeShare 7; GE Healthcare, Chicago, IL, USA). Detailed reconstruction was generated using a mono voltage of 70 keV and the thinnest possible slice thickness of 0.625 mm. The image resolution was 512 pixels × 512 pixels, with each pixel size measuring 0.683594 mm × 0.683594 mm. The resulting images were saved in DICOM format. Imaging was conducted at a 16-bit quality resolution, and gray levels were displayed in Hounsfield units (HUs). The initial image evaluation was performed using a bone window set at a level of +350 and a width of 2000. None of the horses showed radiological signs of paranasal sinus disease during the initial screening, and no missing teeth were observed in any of the horses.

### 2.3. CT Images’ Segmentation and Morphometric Measures’ Extraction

Eight sinuses (FS, DCS, VCS, RMS, CMS, SS, PS, and MCS) were considered on both the right and left sides of each head during segmentation. The extent of the FS was defined dorsally from the frontomaxillary aperture and dorsolaterally from the conchofrontal aperture. The extent of the DCS was defined ventromedially from the conchofrontal aperture. The extent of the VCS was defined medially from the conchomaxillary aperture. The extent of the RMS was defined laterally from the conchomaxillary aperture, laterally from the RMS’s entrance into the nasomaxillary aperture, and rostrally from the maxillary septum. The extent of the CMS was defined caudally from the maxillary septum, laterally from the CMS’s entrance into the nasomaxillary aperture, ventrally from the frontomaxillary aperture, and rostrodorsally from the sphenopalatinal aperture. The extent of the SS was defined caudoventrally from the sphenopalatinal aperture and rostrally from the optic groove. The extent of the PS was defined caudally from the optic groove. The extent of the MCS was defined medially from the so-called ‘ethmoidomaxillary aperture’, which, according to the literature, lacks a proper scientific name [2,4,5,6,13,27,28].

**Figure 1:**
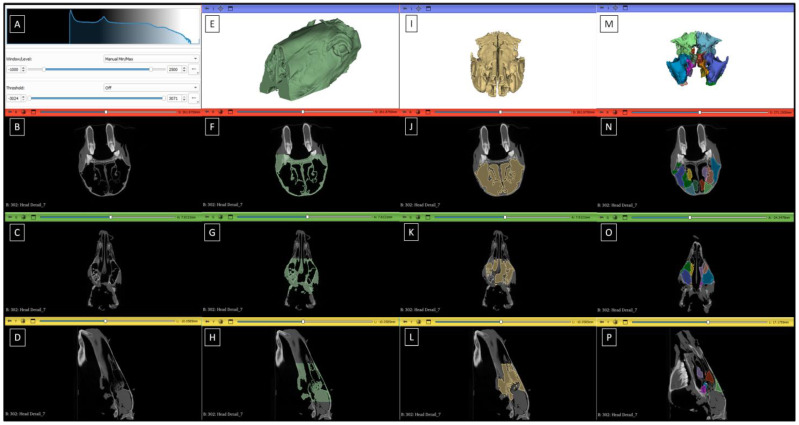
The steps of the 3D segmentation of equine paranasal sinuses. The first step (**A**–**D**) is represented by the gray level mapping settings (**A**), axial plane (**B**), coronal plane (**C**), and sagittal plane (**D**) of raw CT images. The second step (**E**–**H**) is represented by the volume rendering (**E**), axial plane (**F**), coronal plane (**G**), and sagittal plane (**H**) of CT images with segmented tissue with a relative density ≥ 700 HU. The third step (**I**–**L**) is represented by the volume rendering (**I**), axial plane (**J**), coronal plane (**K**), and sagittal plane (**L**) of CT images with segmented tissue with a relative density < 700 HU. The fourth step (**M**–**P**) is represented by the volume rendering (**M**), axial plane (**N**), coronal plane (**O**), and sagittal plane (**P**) of CT images with manually segmented sinuses.

Semiautomated segmentation and morphometric measurements were conducted using Slicer 3D 5.6.0 software [29]. The DICOM files were imported into the Slicer 3D software. In the first step, gray level mapping was set between −1000 HU and 2500 HU, and the histogram was normalized for all datasets (Figure 1A–D). In the second step, tissues in the study area with a relative density ≥ 700 HU were segmented (Figure 1E–H). In the third step, the sinus space and nasal cavity space with a relative density < 700 HU were segmented (Figure 1I–L). In the fourth step, individual sinuses were manually separated from the sinus space and nasal cavity space using previously described ranges (Figure 1M–P). For each segmented sinus, the following morphometric measures were extracted: volume (mm^3^), surface area (mm^2^), and average relative density (HU). Raw volume and surface area data were converted to cm^3^ and cm^2^, respectively. The entire procedure was conducted in accordance with Brinkschulte et al. [9] and Köhler et al. (2021) [13].

### 2.4. Statistical Analysis

The obtained numerical data representing the eight sinuses were presented for both the right and left sides independently. Data series were tested for their distribution using a Shapiro–Wilk normality test. As at least one data series for each extracted morphometric measure was not normally distributed, the numerical data were presented on plots using the median ± ranges (lower and upper percentile; minimum and maximum values), as well as in tables using the median ± ranges (lower and upper percentile). The significance level was established as *p* < 0.05.

First, axial symmetry was tested by comparing the extracted morphometric measures between the left and right sides using an unpaired t-test with Welch’s correction or the Mann–Whitney test, depending on the data distribution.

Next, data series from the right and left sides were grouped into the following location-related groups: FS, DCS, VCS, RMS, CMS, SS, PS, and MCS. Grouped data series were again tested for distribution using a Shapiro–Wilk normality test. Then, the extracted morphometric measures were compared between location-related groups. When all data series were normally distributed, the location-related groups were compared using a one-way ANOVA summary followed by Holm–Sidak’s multiple comparisons test. When at least one data series was not normally distributed, the Kruskal–Wallis test, followed by Dunn’s multiple comparisons test, was used.

Subsequently, within each location-related group, the data series were subdivided into young, middle-aged, and old horse groups. Each age-related group contained data from both the right and left sides. Subgrouped data series were again tested for their distribution using a Shapiro–Wilk normality test. Then, the extracted morphometric measures were compared between age-related groups. When all data series were normally distributed, the age-related groups were compared using a one-way ANOVA summary followed by Holm–Sidak’s multiple comparisons test. When at least one data series was not normally distributed, the Kruskal–Wallis test, followed by Dunn’s multiple comparisons test, was used. The comparison between location-related groups was repeated for each age-related group, respectively.

Finally, datasets representing the three morphometric measures and three age-related groups were clustered using the K-means clustering algorithm. A Python scikit-learn module (https://scikit-learn.org/stable/modules/generated/sklearn.cluster.KMeans.html#sklearn.cluster.KMeans; accessed on 12 April 2024) in the Python 3.11 scripting language was used. Each morphometric measure was scaled to a given range between zero and one using MinMaxScaler from a Python scikit-learn module with integrated classical machine learning algorithms (https://scikit-learn.org/stable/modules/generated/sklearn.preprocessing.MinMaxScaler.html; accessed on 12 April 2024). Principal components of the morphometric measures were calculated using principal component analysis (PCA). The protocol was repeated for four combinations of morphometric measures: volume/surface area/relative density; volume/surface area; volume/relative density; and surface area/relative density. For each combination, classification metrics (recall, precision, accuracy, and F1–score) were calculated.

## 3. Results

### 3.1. Axial Symmetry of the Sinuses

The volume, surface area, and relative density extracted from each paranasal sinus did not differ between the right and left sides. Therefore, the morphometric data were grouped for both sides and are presented in Supplementary Tables S1–S3, respectively.

### 3.2. Location-Related Differences in Morphometric Measures

**Figure 2:**
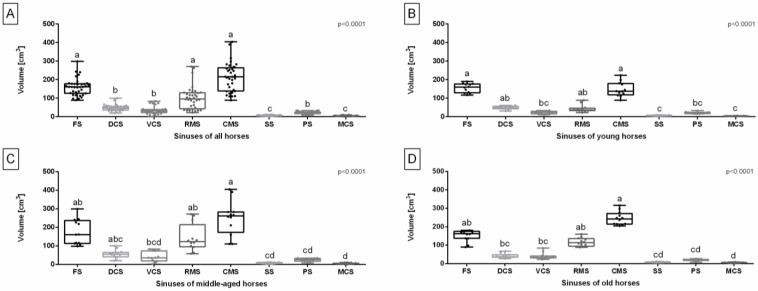
Volume of the equine paranasal sinuses (frontal sinus (FS), dorsal conchal sinus (DCS), ventral conchal sinus (VCS), rostral maxillary sinus (RMS), caudal maxillary sinus (CMS), sphenoid sinus (SS), palatine sinus (PS), and middle conchal sinus (MCS)) of all horses (**A**), young horses (**B**), middle-aged horses (**C**), and old horses (**D**). Data in box plots are represented by lower quartile, median, and upper quartile, whereas whiskers represent minimum and maximum values. Lowercase letters indicate differences between sinuses. Points represent single individuals. The significance level was established as *p* < 0.05.

Considering all studied horses, the volume of FS, RMS, and CMS was higher than the volume of the remaining sinuses. Additionally, the volume of DCS, VCS, and PS was higher than the volume of SS and MCS (Figure 2A).

In the young horses group, the volume of the FS and CMS was higher than the volume of the VCS, SS, PS, and MCS. Furthermore, the volume of the DCS and RMS was higher than the volume of the SS and MCS (Figure 2B). In the middle-aged horses group, the volume of the CMS was higher than that of the VCS, SS, PS, and MCS. The volume of the FS and RMS was also higher than the volume of the SS, PS, and MCS, and the volume of the DCS was higher than the volume of the MCS (Figure 2C). In the old horses group, the volume of the CMS was higher than the volume of the DCS, VCS, SS, PS, and MCS. Additionally, the volume of the FS and RMS was higher than the volume of the SS, PS, and MCS, and the volume of the DCS and VCS was higher than the volume of the MCS (Figure 2D).

**Figure 3:**
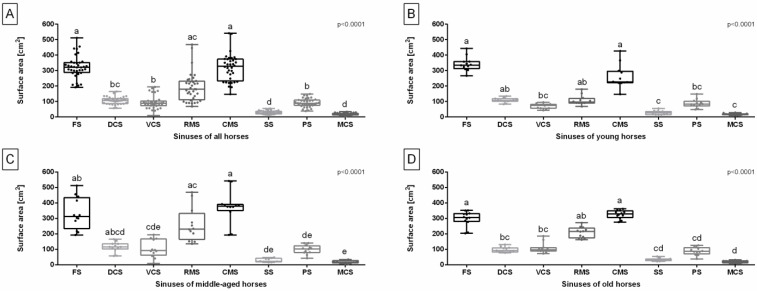
Surface area of the equine paranasal sinuses (frontal sinus (FS), dorsal conchal sinus (DCS), ventral conchal sinus (VCS), rostral maxillary sinus (RMS), caudal maxillary sinus (CMS), sphenoid sinus (SS), palatine sinus (PS), and middle conchal sinus (MCS)) of all horses (**A**), young horses (**B**), middle-aged horses (**C**), and old horses (**D**). Data in box plots are represented by lower quartile, median, and upper quartile, whereas whiskers represent minimum and maximum values. Lowercase letters indicate differences between sinuses. Points represent single individuals. The significance level was established as *p* < 0.05.

Considering all studied horses, the surface area of the FS and CMS was higher than the surface area of the DCS, VCS, SS, PS, and MCS. Additionally, the surface area of the RMS was higher than the surface area of the VCS, SS, PS, and MCS. The surface area of the DCS, VCS, and PS was also higher than the surface area of the SS and MCS (Figure 3A). In the young horses group, the surface area of the FS and CMS was higher than the surface area of the VCS, SS, PS, and MCS. Moreover, the surface area of the DCS, VCS, and RMS was higher than the surface area of the SS and MCS (Figure 3B). In the middle-aged horses group, the surface area of the CMS was higher than the surface area of the VCS, SS, PS, and MCS. The surface area of the FS and RMS was higher than the surface area of the SS, PS, and MCS. Additionally, the surface area of the DCS and VCS was higher than the surface area of the MCS (Figure 3C). In the old horses group, the surface area of the FS and CMS was higher than the surface area of the DCS, VCS, SS, PS, and MCS. The surface area of the RMS was higher than the surface area of the SS, PS, and MCS. Furthermore, the surface area of the DCS and VCS was higher than the surface area of the MCS (Figure 3D).

**Figure 4:**
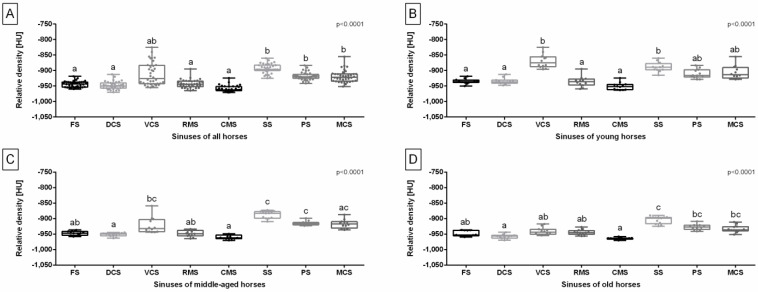
Relative density of the equine paranasal sinuses (frontal sinus (FS), dorsal conchal sinus (DCS), ventral conchal sinus (VCS), rostral maxillary sinus (RMS), caudal maxillary sinus (CMS), sphenoid sinus (SS), palatine sinus (PS), and middle conchal sinus (MCS)) of all horses (**A**), young horses (**B**), middle-aged horses (**C**), and old horses (**D**). Data in box plots are represented by lower quartile, median, and upper quartile, whereas whiskers represent minimum and maximum values. Lowercase letters indicate differences between sinuses. Points represent single individuals. The significance level was established as *p* < 0.05.

Considering all studied horses, the relative density of the FS, DCS, RMS, and VCS was lower than the relative density of the SS, PS, and MCS (Figure 4A). In the young horses group, the relative density of the FS, DCS, RMS, and CMS was lower than the relative density of the VCS and SS (Figure 4B). In the middle-aged horses group, the relative density of the DCS and CMS was lower than the relative density of the VCS, SS, and PS. Additionally, the relative density of the FS and RMS was lower than the relative density of the SS and PS (Figure 4C). In the old horses group, the relative density of the DCS and CMS was lower than the relative density of the SS, PS, and MCS. Moreover, the relative density of the FS, VCS, and RMS was lower than the relative density of the SS (Figure 4D).

### 3.3. Age-Related Differences in Morphometric Measures

**Figure 5:**
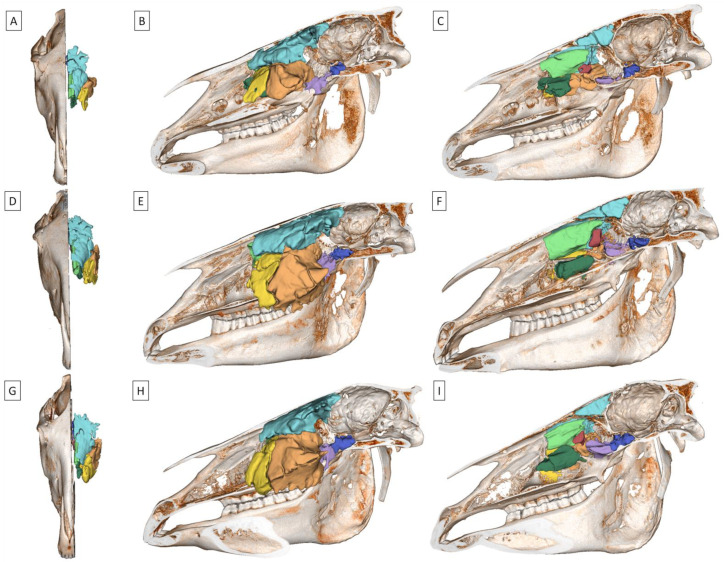
3D models of the segmented equine paranasal sinuses (frontal sinus (FS; light blue), dorsal conchal sinus (DCS; light green), ventral conchal sinus (VCS; dark green), rostral maxillary sinus (RMS; yellow), caudal maxillary sinus (CMS; orange), sphenoid sinus (SS; purple), palatine sinus (PS; dark blue), and middle conchal sinus (MCS; dark red)) of young horses (**A***–***C**), middle-aged horses (**D***–***F**), and old horses (**G**–**I**). The segmented sinuses of the right side of the head are visible from a dorsal (**A**,**D**,**G**), lateral (**B**,**E**,**H**), and medial (**C**,**F**,**I**) view.

To facilitate a comparison of the age-related groups, the size, range, and location of the studied paranasal sinuses were visualized using 3D models, as seen in Figure 5. The volume of the VCS, RMS, and CMS was higher in the middle-aged and old horses groups compared to the young horses group. However, no differences were found for the FS, DCS, SS, PS, and MCS (Table 1).

The surface area of the VCS, RMS, and CMS was higher in the old horses group than in the young horses group; similarly, the surface area of the RMS and CMS was higher in the middle-aged horses group than in the young horses group. However, no differences were found for the FS, DCS, SS, PS, and MCS (Table 2).

The relative density of the FS, DCS, VCS, CMS, SS, PS, and MCS was lower in the old horses group than in the young horses group. Additionally, the relative density of the FS, DCS, VCS, and CMS was lower in the middle-aged horses group than the young horses group. No differences were found for the RMS (Table 3).

### 3.4. Computer-Aided Identification of Age-Related Variations

**Figure 6:**
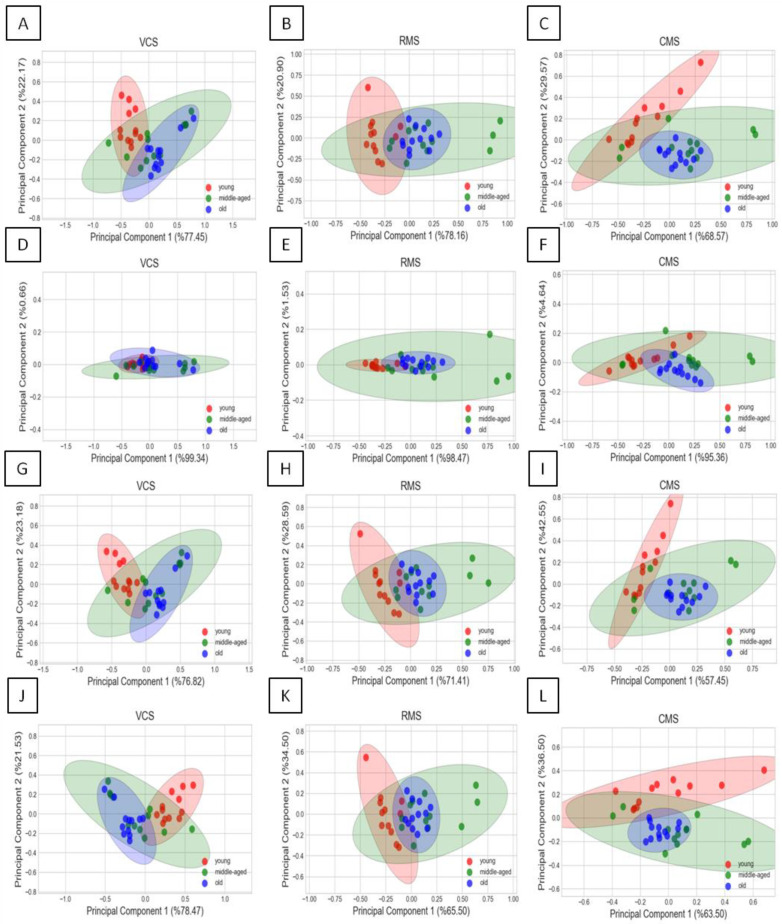
Three age-related classes (young horses, middle-aged horses, old horses) extracted from the morphometric measures of the volume of the ventral conchal sinus (VCS) (**A**,**D**,**G**,**J**), rostral maxillary sinus (RMS) (**B**,**E**,**H**,**K**), and caudal maxillary sinus (CMS) (**C**,**F**,**I**,**L**). Principal components are calculated for the four combinations of morphometric measures: volume, surface area, and relative density (**A**–**C**); volume and surface area (**D**–**F**); volume and relative density (**G**–**I**); and surface area and relative density (**J**–**L**).

For the three paranasal sinuses (VCS, RMS, CMS) that showed age-dependent differences in their morphometric measures, a PCA was performed, as shown in Figure 6. Red points represented young horses, blue points represented middle-aged horses, and green points represented old horses in the scaled space created based on three (Figure 6A–C) or two (Figure 6D–L) morphometric measures.

The accuracy of the VCS’s classification was higher for the volume/surface area measures combination than for other combinations; however, in none of the considered combinations was the accuracy ≥ 0.5 (Table 4). The accuracy of the RMS’s classification was the highest for the volume/relative density measures combination and relatively high for the surface area/relative density measures combination. However, only for the volume/relative density measures combination was the accuracy ≥ 0.5 (Table 5). The accuracy of the CMS’s classification was the highest for the volume/surface area/relative density measures combination and relatively high for the surface area/relative density and volume/relative density measures combinations. Moreover, for both the volume/surface area/relative density and surface area/relative density measures combinations, the accuracy was ≥0.5 (Table 6).

With accuracy values of 0.72 for the RMS, 0.67 for the CMS, and 0.31 for the VCS, the possibility of the age-related clustering of CT-based 3D images of equine paranasal sinuses was confirmed for the RMS and CMS and disproved for VCS.

## 4. Discussion

This study demonstrated that the individual segmentation of the eight paranasal sinuses in horses allows for the extraction of morphometric measurements, which vary specifically depending on the location of the sinus and the age of the horse. However, the novelty of the current study lies primarily in advancing the clinical utility of equine sinus segmentation by extracting three morphometric features rather than one and using them as input for computer-aided classifiers. The results of the current research indicate which morphometric measures affected the accuracy of the classification of segmented sinuses, considering the two most important variables influencing these numerical values: the location of the sinus and the age of the horse. This preliminary methodology may be used in further research on the computer-aided segmentation of equine paranasal sinuses.

The current results are consistent with previous reports indicating that the maxillary sinus is the largest paranasal sinus in adult horses [6]. Considering that the maxillary sinus is divided by a thin maxillary septum into the CMS and RMS [1], the largest volume compartment, as recently and currently reported, is the CMS [9,10,14], followed by the FS [9]. The smallest volume, as recently and currently reported, is the MCS [9]. One study suggested that the FS is the largest and the MCS the smallest sinus in foals, but this statement was not statistically supported [3].

The sinus volume reported in this study was within the ranges previously reported for Warmblood [9] and Thoroughbred [9,10] horses. However, it was larger than the sinus volume reported for smaller horses such as adult Arabian horses [14], foal Arabian horses [3], and adult miniature horses [13]. This is consistent with the variations in the conformation [30], shape [31,32], and size [33] of the skull of various horse breeds. The general proportion between the volume of individual sinuses [3] was considered, although, in some previous studies, the SS and PS were assessed together as the SPS [9,10,13], and one study considered the FS and DCS together as the CFS [14]. Additionally, the sinuses may be grouped into two functional systems: the rostral sinus system and the caudal sinus system. The rostral sinus system contains the RMS and VCS, whereas the caudal sinus system contains the FS, DCS, CMS, SS, PS, and MCS; both systems are completely separated by the maxillary septum [8,9]. This functional approach is crucial for drainage into the nasal cavity [5,6,9]; however, it seems to have limited usefulness in the development of computer-aided classifiers. Noteworthy in this study, and similar to previous volumetric measurements performed on healthy horses’ heads, is the fact that no differences were found between the volumes of the sinuses on the right and left sides [9,10,13]. The consistency of the recent and present results indicates the effectiveness of the segmentation performed.

Age-dependent differences in the volume of the paranasal sinuses, assessed by CT, were reported in two publications [9,10]. The first publication reports a trend of increased sinus volume with age, but not specific differences in morphometric measures [9], whereas the second publication provides more detailed data [10]. The authors observed that the DCS was larger than the FS in middle-aged (6–15 years of age) and old (>15 years of age) horses but not in young (<6 years of age) horses [10]. In contrast, in this study, the volume of the DCS was lower than that of the FS in old horses, and there were no differences in the DCS and FS’s volume in young and middle-aged horses. The current results are consistent with a previous report describing the FS as the second biggest sinus in adult horses [9]. Moreover, a previous study reported that the DCS is larger than the VCS regardless of age-related group [10], whereas, in this study, no differences in volume and surface area were found. These differences may result from the ambiguous boundary between the DCS and FS, which is hard to define due to the large conchofrontal aperture, so, in some publications, they are defined as one CFS [4,5]. This explanation seems to be justified because in both previous studies [9,10] and this study, the FS and DCS combined into the CFS are very similar in volume.

Importantly, both a recent study [10] and this study agree that the volume of the VCS, RMS, and CMS increases age-dependently, while the volume of the FS, SP, PS (combined into SPS [10]), and MCS (named ES [10]) does not change with horse age. One may observe that the VCS, RMS, and CMS contain the dental alveoli of cheek teeth and would therefore be expected to increase in volume with dental eruption [10]. The alveoli of the second and third maxillary premolar teeth (Triadan 106/206 and 107/207 [33]) lie in the maxillary bone, the fourth maxillary premolar and first maxillary molar teeth (Triadan 108/208 and 109/209 [33]) lie within the RMS/VCS, and the second and third maxillary molar teeth (Triadan 110/210 and 111/211 [33]) lie within the CMS in young-adult horses [6,7,34]. In young horses (<5 years of age), the RMS and CMS are largely filled with embedded parts of the four maxillary cheek teeth (Triadan 108/208–111/211 and 208–211 [1]) [7]. With prolonged eruption, the reserve crown length of the maxillary cheek teeth decreases with age by 2.2 to 4.7 mm each year [10,35,36]. The rate of reserve crown shortening, and the associated increase in related sinus volume, decreases with age, so the rate of clinical crown eruption slows as horses age [35,36]. Moreover, cheek teeth drift with age in a rostral direction, which is contributed to by the continued eruption of rostrally angulated clinical crowns and additionally increases the volume of the related sinuses [10].

### 4.1. Limitations

The main limitation of this study primarily results from the small sample size. Previous studies on the segmentation of equine paranasal sinuses were conducted on 5 isolated heads [3], 10 isolated heads [14], 12 scans of either ponies or isolated pony heads [13], 18 isolated heads [9], 18 horses [23], or 30 isolated heads [10]. The small sample size is understandable due to the time-consuming nature of the method and the need to obtain high-quality clinical data. Because of the complex anatomy of the studied area [13], our semi-automated segmentation protocol requires CT images to be visualized slide by slide and manually corrected as needed. Thus, the segmentation of one head took about 8–12 h, making it challenging to incorporate into an everyday workflow [9]. Hence, one of the next steps is to develop automatic segmentation protocols supported by artificial intelligence algorithms that could provide accurate morphometric measurements in a much shorter time. Therefore, the current preliminary methodology requires expansion with subsequent steps for computer-aided segmentation and repetition on a larger group of horses. Particularly useful would be a group similar to the one used in the study of tooth size variability [36], which enabled the assessment of linear age-related relations.

In clinical applications, the primary interest lies in the affected sinuses [12,20,23,37]. In the course of most diseases, such as infectious sinusitis, dental sinusitis, traumatic sinusitis, sinus cyst, progressive ethmoid hematoma, and sinus neoplasia, the swelling of the sinus mucous membrane and the accumulation of exudate can significantly alter the aeration and volume of the affected sinuses [12,16,17,18]. Since this study evaluated clinically and radiologically healthy isolated heads, the uncritical clinical usefulness of computer-assisted classifiers needs to be tested under clinical conditions. Therefore, further research should include the application of the proposed methodology to a larger group of horses, and particularly clinical cases that encompass both healthy and affected sinuses. A study of this nature, accounting for the age- and combination-dependent classification variability demonstrated here, will provide new insights into the clinically important development of computer-assisted imaging diagnoses of equine paranasal sinus diseases.

### 4.2. Further Directions

One may observe that none of the recent CT-based studies on paranasal sinus segmentation investigated the surface area and relative density of the segmented sinuses [3,9,10,13,14]. One study was conducted solely the for preoperative visualization of the sinuses without any morphometric measurements, including volumetric ones [23]. While one publication declared both volume and surface area [3], only the volume was numerically reported for individual sinuses, with the surface area reported collectively for one side of the sinus system. Interestingly, these two previously unconsidered morphometric measures also demonstrated location-related and age-related variability. While this is not surprising for the surface area, as a volume-dependent variable [3], it is noteworthy for the relative density. Moreover, among the studied morphometric measures, relative density rather than surface area was identified as the principal component affecting classification accuracy. Thus, a high accuracy of the RMS and CMS’s classification was evidenced for a combination of volumetric features and relative density.

In recent studies, each volumetric measure was extracted using different image processing software; however, the data extraction protocol was generally consistent. Two studies utilized professional, fully-paid software: MIMICS (Materialise’s Interactive Medical Image Control System, ver: 12.01, Materialise, Leuven, Belgium) [3] and Osirix DICOM viewer (Pixmeo SARL, Bernex, Switzerland) [10]. In two other studies, free trial software, which required payment for the full version, was used: Amira 5.3.3R (Amira 5.3.3R, Visage Imaging, Berlin, Germany) [9] and RadiAnt DICOM Viewer (Medixant, Poznań, Poland) [14]. Recently, only one study employed the free CIBC Seg3D 2 Segmentation software (NIH Center for Integrative Biomedical Computing (CIBC), Salt Lake City, UT, USA) [13]. Therefore, this study represents the second instance of the successful utilization of freely available software for equine paranasal sinus segmentation. As at least nine different commercial/open source software packages are available for DICOM image segmentation [38], and this number continues to grow, this direction of research and its broader clinical application may develop dynamically.

Previous publications have described CT-based segmentation as less accessible due to the limited use of CT in equine clinics [3,23]. However, more recent reports suggest an increasing use of equine CT [10]. With recent equipment advancements and modifications allowing for fan-beam CT use in standing sedated horses [39,40], high-resolution standing sedated CT of the equine head is becoming more readily available. Therefore, the use of free software for semiautomated sinus segmentation and the development of automatic segmentation protocols are desirable and necessary directions for further research. Considering the potential practical applications of this protocol, one can observe that 3D volume renderings provide additional information about the relative position of the paranasal sinus compartments and the relation of the paranasal sinuses to other structures of the head [9]. Therefore, quickly extracted and accurate morphometric data may contribute to everyday workflows, aiding in the understanding of individual clinical and radiographic findings [9], evaluating sinus disease [12,37], and planning potential surgical interventions [20,23].

## 5. Conclusions

The proposed freely available CT image segmentation protocol allows for the extraction of morphometric measures characteristic of individual equine paranasal sinuses. By expanding existing volumetric measurements of the sinuses to include additional morphometric measures such as surface area and relative density, this approach offers valuable location-related and age-related data. These data can be combined to improve the accuracy of computer-aided classifiers. For the RMS and CMS, the two main sinuses that increase in volume as cheek teeth eruption progresses with age, clustering analysis achieved the highest classification accuracy using combinations of volume/relative density and volume/surface area/relative density, respectively.

## Figures and Tables

**Table 1:** Volume (cm^3^) (median and range (lower percentile; upper percentile)) of the equine paranasal sinuses (frontal sinus (FS), dorsal conchal sinus (DCS), ventral conchal sinus (VCS), rostral maxillary sinus (RMS), caudal maxillary sinus (CMS), sphenoid sinus (SS), palatine sinus (PS), and middle conchal sinus (MCS)) as compared between the young, middle-aged, and old horses groups. The significance level was established as *p* < 0.05.

	FS	DCS	VCS	RMS	CMS	SS	PS	MCS
Young horses	159.4 ^a^	47.5 ^a^	23.4 ^a^	36.0 ^a^	137.3 ^a^	5.0 ^a^	19.1 ^a^	3.5 ^a^
	(128.6; 177.0)	(43.6; 55.1)	(15.2; 28.5)	(32.9; 46.5)	(117.7; 178.9)	(3.7; 8.1)	(16.0; 23.7)	(2.9; 4.4)
Middle-aged horses	160.6 ^a^	53.9 ^a^	35.5 ^b^	122.4 ^b^	261.5 ^b^	4.6 ^a^	25.2 ^a^	3.2 ^a^
	(113.0; 236.7)	(40.9; 64.9)	(18.9; 73.2)	(94.9; 213.4)	(172.1; 282.5)	(2.8; 10.6)	(16.8; 31.8)	(2.0; 6.3)
Old horses	162.6 ^a^	36.8 ^a^	32.1 ^b^	113.5 ^b^	242.3 ^b^	6.0 ^a^	21.0 ^a^	3.9 ^a^
	(137.1; 175.3)	(34.6; 47.5)	(30.6; 40.9)	(94.3; 136.0)	(214.7; 272.0)	(5.0; 8.9)	(17.6; 22.0)	(2.4; 6.3)
*p*	0.9	0.2	0.01	<0.0001	0.0003	0.3	0.4	0.4

^a,b^ Lowercase letters indicate differences between groups.

**Table 2:** Surface area (cm^3^) (median and range (lower percentile; upper percentile)) of the equine paranasal sinuses (frontal sinus (FS), dorsal conchal sinus (DCS), ventral conchal sinus (VCS), rostral maxillary sinus (RMS), caudal maxillary sinus (CMS), sphenoid sinus (SS), palatine sinus (PS), and middle conchal sinus (MCS)) as compared between the young, middle-aged, and old horses groups. The significance level was established as *p* < 0.05.

	FS	DCS	VCS	RMS	CMS	SS	PS	MCS
Young horses	335.9 ^a^	106.6 ^a^	75.7 ^a^	96.9 ^a^	226.5 ^a^	26.7 ^a^	83.0 ^a^	16.6 ^a^
	(312.9; 358.0)	(102.2; 118.1)	(56.7; 81.8)	(90.5; 119.0)	(221.2; 295.8)	(15.2; 35.7)	(71.6; 101.5)	(14.5; 20.9)
Middle-aged horses	311.7 ^a^	115.2 ^a^	92.0 ^ab^	230.7 ^b^	378.2 ^b^	22.2 ^a^	102.9 ^a^	17.1 ^a^
	(233.6; 434.4)	(101.2; 135.0)	(61.8; 167.3)	(164.0; 331.2)	(350.2; 390.5)	(18.4; 41.7)	(79.2; 121.8)	(11.4; 26.7)
Old horses	305.7 ^a^	91.0 ^a^	97.1 ^b^	215.4 ^b^	328.0 ^b^	28.8 ^a^	86.9 ^a^	17.8 ^a^
	(279.5; 330.6)	(82.3; 108.8)	(89.3; 109.8)	(174.1; 237.1)	(305.1; 348.3)	(26.3; 37.3)	(69.7; 110.1)	(13.0; 24.5)
*p*	0.4	0.2	0.008	<0.0001	0.004	0.5	0.6	0.9

^a,b^ Lowercase letters indicate differences between groups.

**Table 3:** Relative density (HU) (median and range (lower percentile; upper percentile)) of the equine paranasal sinuses (frontal sinus (FS), dorsal conchal sinus (DCS), ventral conchal sinus (VCS), rostral maxillary sinus (RMS), caudal (maxillary sinus (CMS), sphenoid sinus (SS), palatine sinus (PS), and middle conchal sinus (MCS)) as compared between the young, middle-aged, and old horses groups. The significance level was established as *p* < 0.05.

	FS	DCS	VCS	RMS	CMS	SS	PS	MCS
Young horses	−936 ^a^	−937 ^a^	−875 ^a^	−936 ^a^	−952 ^a^	−890 ^a^	−916 ^a^	−914 ^a^
	(−939; −931)	(−941; −931)	(−888; −856)	(−947; −928)	(−962; −945)	(−897; −878)	(−921; −898)	(−924; −891)
Middle-aged horses	−947 ^b^	−950 ^b^	−932 ^b^	−949 ^a^	−962 ^b^	−883 ^a^	−917 ^a^	−918 ^a^
	(−954; −942)	(−955; −947)	(−942; −903)	(−956; −938)	(−965; −953)	(−899; −878)	(−921; −913)	(−931; −910)
Old horses	−953 ^b^	−957 ^b^	−944 ^b^	−945 ^a^	−964 ^b^	−899 ^b^	−927 ^b^	−936 ^b^
	(−956; −938)	(−963; −955)	(−951; −935)	(−951; −939)	(−967; −963)	(−917; −897)	(−935; −922)	(−940; −926)
*p*	0.001	<0.0001	<0.0001	0.06	0.001	0.004	0.002	0.0012

^a,b^ Lowercase letters indicate differences between groups.

**Table 4:** The identification of age-related variations of the ventral conchal sinus (VCS). The classification metrics (recall, precision, F1–score, accuracy) of K-means clustering into three specific age-related classes (young horses, middle-aged horses, old horses). Metrics are calculated for the four combinations of morphometric measures (volume, surface area, relative density).

Measures	Groups	Recall	Precision	F1	Accuracy
volume/surface area/relative density					
	Young horses	0.00	0.00	0.00	0.11
	Middle-aged horses	0.33	0.67	0.44	
	Old horses	0.00	0.00	0.00	
volume/surface area					
	Young horses	0.58	0.32	0.41	0.31
	Middle-aged horses	0.33	0.67	0.44	
	Old horses	0.00	0.00	0.00	
volume/relative density					
	Young horses	0.00	0.00	0.00	0.11
	Middle-aged horses	0.17	0.14	0.15	
	Old horses	0.17	0.33	0.22	
surface area/relative density					
	Young horses	0.00	0.00	0.00	0.11
	Middle-aged horses	0.17	0.14	0.15	
	Old horses	0.17	0.33	0.22	

**Table 5:** The identification of age-related variations of the rostral maxillary sinus (RMS). The classification metrics (recall, precision, F1–score, accuracy) of K-means clustering into three specific age-related classes (young horses, middle-aged horses, old horses). Metrics are calculated for the four combinations of morphometric measures (volume, surface area, relative density).

Measures	Groups	Recall	Precision	F1	Accuracy
volume/surface area/relative density					
	Young horses	0.08	0.05	0.06	0.11
	Middle-aged horses	0.25	1.00	0.40	
	Old horses	0.00	0.00	0.00	
volume/surface area					
	Young horses	0.08	0.05	0.06	0.08
	Middle-aged horses	0.17	0.15	0.16	
	Old horses	0.00	0.00	0.00	
volume/relative density					
	Young horses	1.00	0.80	0.89	0.72
	Middle-aged horses	0.25	1.00	0.40	
	Old horses	0.92	0.61	0.73	
surface area/relative density					
	Young horses	0.67	0.89	0.76	0.47
	Middle-aged horses	0.75	0.38	0.50	
	Old horses	0.00	0.00	0.00	

**Table 6:** The identification of age-related variations of the caudal maxillary sinus (CMS). The classification metrics (recall, precision, F1–score, accuracy) of K-means clustering into three specific age-related classes (young horses, middle-aged horses, old horses). Metrics are calculated for the four combinations of morphometric measures (volume, surface area, relative density).

Measures	Groups	Recall	Precision	F1	Accuracy
volume/surface area/relative density					
	Young horses	0.83	0.83	0.83	0.67
	Middle-aged horses	0.17	0.67	0.27	
	Old horses	1.00	0.57	0.73	
volume/surface area					
	Young horses	0.25	0.13	0.17	0.14
	Middle-aged horses	0.17	0.18	0.17	
	Old horses	0.00	0.00	0.00	
volume/relative density					
	Young horses	0.50	0.86	0.63	0.42
	Middle-aged horses	0.75	0.43	0.55	
	Old horses	0.00	0.00	0.00	
surface area/relative density					
	Young horses	0.83	0.77	0.80	0.50
	Middle-aged horses	0.67	0.42	0.52	
	Old horses	0.00	0.00	0.00	

## Data Availability

The data presented in this study are available upon request from the corresponding author.

## Supplementary Materials

The following supporting information can be downloaded at https://www.mdpi.com/article/10.3390/s24113538/s1, Table S1: Volume of the equine paranasal sinuses extracted from the right and left side of head, as well as grouped for both sides. Table S2: Surface area of the equine paranasal sinuses extracted from the right and left side of head, as well as grouped for both sides. Table S3: Relative density of the equine paranasal sinuses extracted from the right and left side of head, as well as grouped for both sides.
