# Supplementary material for: Three-Dimensional Segmentation of Equine Paranasal Sinuses in Multidetector Computed Tomography Datasets: Preliminary Morphometric Assessment Assisted with Clustering Analysis
Source: Sensors (Basel). 2024 May 30;24(11):3538. doi: 10.3390/s24113538 (PMC11175080; doi:10.3390/s24113538)
Supplement: Supplementary file 1 [file sensors-24-03538-s001.zip › sensors-3009147-supplementary.pdf]

Supplementary Data for

Three-Dimensional Segmentation of Equine Paranasal Sinuses in Multidetector Computed Tomography Datasets: Preliminary Morphometric Assessment Assisted with Clustering Analysis

Marta Borowska [1,*], Paweł Lipowicz [1], Kristina Daunoravičienė [2], Bernard Turek [3], Tomasz Jasiński [3], Jolanta Pauk [1], and Małgorzata Domino [3,*]

1 Institute of Biomedical Engineering, Faculty of Mechanical Engineering, Białystok University of Technology, 15–351 Bialystok, Poland; p.lipowicz@pb.edu.pl (P.L.); j.pauk@pb.edu.pl (J.P.)
2 Department of Biomechanical Engineering, Vilnius Gediminas Technical University, 03224 Vilnius, Lithuania; kristina.daunoraviciene@vilniustech.lt (K.D.)
3 Department of Large Animal Diseases and Clinic, Institute of Veterinary Medicine, Warsaw University of Life Sciences, 02–787 Warsaw, Poland; bernard_turek@sggw.edu.pl (B.T.); tomasz_jasinski@sggw.edu.pl (T.J.)
* Correspondence: m.borowska@pb.edu.pl (M.B.); malgorzata_domino@sggw.edu.pl (M.D.)

Table S1. Volume (cm^3) (mediane and range (lower percentile; upper percentile)) of the equine paranasal sinuses (frontal sinus (FS), dorsal conchal sinus (DCS), ventral conchal sinus (VCS), rostral maxillary sinus (RMS), caudal maxillary sinus (CMS), sphenoid sinus (SS), palatine sinus (PS), and middle conchal sinus (MCS)) extracted from the right and left side of head as well as grouped for both sides. The significance level was established as $p < 0.05$.

	FS	DCS	VCS	RMS	CMS	SS	PS	MCS
Right	158.8	45.6	31.7	97.3	213.1	4.8	21.2	3.0
side	(116.7; 175.2)	(36.1; 55.8)	(21.8; 36.5)	(46.5; 130.4)	(134.6; 262.0)	(3.4; 8.7)	(16.2; 24.2)	(2.2; 4.5)
Left	166.0	47.5	29.2	95.7	218.7	5.7	21.3	4.1
side	(134.9; 178.4)	(35.5; 56.5)	(21.1; 41.0)	(36.6; 130.6)	(142.8; 268.7)	(4.7; 9.3)	(18.3; 26.3)	(2.6; 6.0)
Both	160.5	46.4	31.4	96.4	215.0	5.5	21.3	3.5
sides	(126.1; 177.6)	(36.0; 55.7)	(22.5; 38.4)	(43.6; 129.8)	(139.6; 264.6)	(4.3; 8.9)	(17.0; 25.2)	(2.5; 5.7)
p	0.3	0.8	0.8	0.9	0.9	0.3	0.4	0.3

Table S2. Surface area (cm^2) (mediane and range (lower percentile; upper percentile)) of the equine paranasal sinuses (frontal sinus (FS), dorsal conchal sinus (DCS), ventral conchal sinus (VCS), rostral maxillary sinus (RMS), caudal maxillary sinus (CMS), sphenoid sinus (SS), palatine sinus (PS), and middle conchal sinus (MCS)) extracted from the right and left side of head as well as grouped for both sides. The significance level was established as $p < 0.05$.

	FS	DCS	VCS	RMS	CMS	SS	PS	MCS
Right	308.4	106.1	89.0	176.2	320.6	24.7	90.8	14.8
side	(279.7; 332.3)	(88.7; 116.1)	(72.8; 103.7)	(119.0; 242.0)	(227.1; 367.7)	(17.7; 36.5)	(68.7; 105.8)	(12.3; 22.3)
Left	332.3	106.8	81.6	191.0	340.5	31.9	83.7	18.2
side	(301.5; 369.8)	(85.7; 122.5)	(69.3; 103.5)	(103.1; 226.1)	(227.1; 367.7)	(22.1; 39.6)	(79.9; 120.3)	(13.6; 25.5)
Both	322.4	106.4	88.9	179.7	328.0	27.0	88.9	16.6
sides	(288.0; 351.3)	(86.9; 118.7)	(72.7; 102.5)	(112.2; 231.6)	(233.0; 374.2)	(19.7; 37.5)	(75.8; 109.2)	(13.2; 23.9)
p	0.2	0.8	0.6	0.8	0.7	0.2	0.6	0.4

Table S3. Relative density (HU) (mediane and range (lower percentile; upper percentile)) of the equine paranasal sinuses (frontal sinus (FS), dorsal conchal sinus (DCS), ventral conchal sinus (VCS), rostral maxillary sinus (RMS), caudal maxillary sinus (CMS), sphenoid sinus (SS), palatine sinus (PS), and middle conchal sinus (MCS)) extracted from the right and left side of head as well as grouped for both sides. The significance level was established as $p < 0.05$.

	FS	DCS	VCS	RMS	CMS	SS	PS	MCS
Right side	-946 (-953; -937)	-950 (-955; -940)	-924 (-942; -884)	-944 (-953; -937)	-962 (-965; -957)	-896 (-903; -880)	-920 (-924; -913)	-919 (-934; -911)
Left side	-940 (-955; -938)	-949 (-958; -940)	-926 (-943; -978)	-945 (-953; -937)	-962 (-965; -952)	-897 (-900; -885)	-920 (-926; -910)	-925 (-935; -910)
Both sides	-943 (-953; -937)	-950 (-957; -940)	-926 (-942; -883)	-944 (-952; -934)	-962 (-965; -952)	-897 (-900; -883)	-920 (-925; -913)	-921 (-934; -911)
p	0.8	0.9	0.9	0.9	0.7	0.9	1.0	0.9
